# Commentary: Investigating the Effects of Anger and Guilt on Unethical Behaviour: A Dual-Process Approach

**DOI:** 10.3389/fpsyg.2017.00159

**Published:** 2017-02-08

**Authors:** Maria Serena Panasiti, Giorgia Ponsi

**Affiliations:** ^1^Department of Psychology, University of Rome “Sapienza”Rome, Italy; ^2^Social and Cognitive Neuroscience Laboratory, IRCCS Fondazione Santa LuciaRome, Italy; ^3^Department of Social Psychology and Social Neuroscience, Institute of Psychology, University of BernBern, Switzerland

**Keywords:** morality and unethical behavior, deception, deliberation, honesty, intrinsic/extrinsic goals, personality

Research provides important evidences about the role of emotions in a wide range of judgments, including moral decisions (Haidt, 2003; Teper et al., 2015). Recent findings show that real compared to hypothetical moral decisions brings about higher physiological activity (Teper et al., 2011). In agreement, studies on the autonomic correlates of deception reported: (i) higher skin conductance (Coricelli et al., 2010); (ii) increased pupil dilation (Hochman et al., 2016); (iii) higher regulation of sympathetic activity before lying for a self-gain when reputation is at risk (Panasiti et al., 2016).

However, there is little evidence regarding the influence of discrete emotions on dishonesty. Notably, exceptions are the few studies reporting that inducing envy (Moran and Schweitzer, 2005; Gino and Pierce, 2009) or anxiety (Kouchaki and Desai, 2015) enhances deception, while inducing anger or fear oppositely influences hypothetical ethical decisions (Kligyte et al., 2013).

In their recent paper, Motro et al. (2016) made considerable advances in the literature, by reporting that induction of anger and guilt enhances and decreases deception, respectively. Crucially, they also report that the influence of these emotions on deception is mediated by the increment of impulsive thinking in the case of anger and by the enhancement of deliberate thinking in the case of guilt. In this commentary, we propose an additional explanation of their findings that aims at connecting the authors' results with those of other important studies about deception.

On the one hand, Motro's results nicely fit with studies showing that guilt induction reduces cheating when experiencing physical weights (Kouchaki et al., 2014) and that anger promotes deception by reducing empathy and enhancing self-interest (Yip and Schweitzer, 2016). Also developmental research shows that in 4- and 8-years old children, anger enhances immoral (aggressive) behavior, and that this increment is mitigated by children's ability to anticipate guilt (Colasante et al., 2016).

Moreover, the mediation of deliberate vs impulsive thinking supports the Theory of “Deliberate Honesty” according to which, when deception is tempting, dishonesty is the immediate choice while honesty would require reflection (Bereby-Meyer and Shalvi, 2015).

On the other hand, recent theories posit that any choice could be impulsive or deliberate depending on the value-based computation between alternatives (Berkman et al., 2016). Accordingly, the Self-Concept Maintenance Hypothesis (Mazar et al., 2008) proposes that deciding whether to deceive involves a conflict between the temptation to dishonestly achieve some benefit (extrinsic goal) and the desire to act according to internalized social norms (intrinsic goal). This conflict is modulated by several variables: dishonesty is enhanced by anonymity (Zhong et al., 2010), time-pressure (Shalvi et al., 2012), monetary priming (Gino and Mogilner, 2014), sense of entitlement (Poon et al., 2013; Schurr and Ritov, 2016), and positive self-concept activation (Khan and Dhar, 2006; Brown et al., 2011); conversely, honesty is enhanced by reading statements that endorse free-will (Vohs and Schooler, 2008), the Ten Commandments (Mazar et al., 2008), a code of honor (Shu et al., 2011), or by the need to safeguard one's own reputation (Panasiti et al., 2011, 2014, 2016).

Here, we propose that anger and guilt might have enhanced the salience of extrinsic (money) vs. intrinsic (honesty) goals, respectively. Anger is triggered when the achievement of one's important extrinsic goal is prevented by somebody or something (Lazarus and Lazarus, 1994; Turner, 2007), or when someone else behaves unfairly (i.e., in a way that prevents others to reach their extrinsic goal; Pillutla and Murnighan, 1996). Differently, guilt is evoked by the awareness that we did not act morally (Sheikh and Janoff-Bulman, 2010) and thus that we did not accomplish an intrinsic goal. These links are supported by findings showing that: (i) anger facilitates attention (He et al., 2013) and gaze-imitation (Terburg et al., 2012) toward rewarding cues and it is associated with reward-related electrocortical activity (Angus et al., 2015); (ii) baseline activity of the insula and guilt aversion promote the achievement of intrinsic social goals (Chang et al., 2011; Baumgartner et al., 2013).

This alternative explanation aims at reconciling the seeming inconsistency between Motro's results and the studies that show how deliberation and impulsivity are not necessary linked to honesty and dishonesty, respectively. It has been showed for example that inducing a deliberate vs. intuitive mindset increases deception (Zhong, 2011) and that honesty is the default choice for most people (Xu and Ma, 2015).

Moreover, Machiavellians and psychopaths who are strategically dishonest and show low sense of guilt, constitute perfect examples of why deliberation (i) is not always triggered by guilt and (ii) is not necessarily associated to honesty. In particular, Machiavellian people feel low sense of guilt for lying (Gozna et al., 2001); have no need to down-regulate their autonomic system before lying (Panasiti et al., 2016), and show no cortical motor inhibition nor reputation effects for lying (Panasiti et al., 2011, 2014). They are also highly strategic (Jones and Paulhus, 2012) and this bring them to a great deal of deception during their everyday life (Kashy and DePaulo, 1996). Similarly, psychopaths are more likely to perform premeditated (deliberate) than impulsive crimes (Swogger et al., 2010), and show a weaker modulation of anticipated guilt in anterior insula (Seara-Cardoso et al., 2016).

Here, we suggest that despite a change in goals' salience might in turn cause a change in the propensity of using deliberate vs. impulsive thinking, style of thinking alone might not be sufficient to modulate participants' ethical behavior. Differently, the crucial modulation might lie in the change of goals' salience itself. This interpretation would explain why (i) manipulative and psychopathic people who are more attracted to extrinsic than intrinsic goals (Mchoskey, 1999) engage in deliberate thinking and yet behave dishonestly; (ii) inducing an impulsive setting without priming extrinsic goals enhances honesty (Zhong, 2011); (iii) honesty becomes the default choice as participants' moral identity increases (Xu and Ma, 2015).

## Author contributions

MSP and GP have made substantial, direct, and intellectual contribution to the work, and approved it for publication.

### Conflict of interest statement

The authors declare that the research was conducted in the absence of any commercial or financial relationships that could be construed as a potential conflict of interest.
